# *Mallotus furetianus* extract protects against ethanol‐induced liver injury via the activation of the cAMP‐PKA pathway

**DOI:** 10.1002/fsn3.1709

**Published:** 2020-06-10

**Authors:** Eri Yoshikawa, Isao Matsui‐Yuasa, Xuedan Huang, Yoshinori Kobayashi, Akiko Kojima‐Yuasa

**Affiliations:** ^1^ Department of Food and Human Health Sciences Graduate School of Human Life Science Osaka City University Osaka Japan; ^2^ Department of Pharmacognosy School of Pharmacy Kitasato University Tokyo Japan

**Keywords:** cAMP‐PKA pathway, Ethanol‐induced liver injury, hepatic stellate cells, *Mallotus furetianus* extract, NADPH oxidase, reactive oxygen species

## Abstract

The protective effects of *Mallotus furetianus* extract (MF) on liver fibrosis induced with ethanol were examined using in vivo and in vitro model. MF treatment suppressed plasma alanine aminotransferase and aspartate aminotransferase activities in ethanol plus carbon tetrachloride (CCl_4_)‐induced cirrhosis rat model. MF also suppressed the increase in type l collagen and α‐smooth muscle actin expression in the livers of ethanol plus CCl4‐induced rat by the maintenance of intracellular glutathione levels. Furthermore, we evaluated the effect of MF on the alcohol‐induced activation of hepatic stellate cells (HSCs), which are responsible for the increased production and deposition of the extracellular matrix in liver injury. Here, we observed the enhancement of the intracellular reactive oxygen species (ROS) levels and the increase in type I collagen and a‐SMA expression in HSCs activated with ethanol. However, the enhanced ROS levels were suppressed with the treatments of MF or diphenyleneiodonium (DPI). Furthermore, the treatment of MF or DPI suppressed the increase in type I collagen and a‐SMA expression activated with ethanol. We also observed that the treatment of MF or LY194002 suppressed the increase in type I collagen expression in HSCs activated with ethanol, suggesting that ethanol induced type I collagen expression via the PI3K‐Akt signaling pathway. On the other hand, the suppression of the synthesis of type I collagen in ethanol and MF‐treated HSCs was inhibited by H‐89. From these results, MF may suppress the increase in the activity of NADPH oxidase in HSCs activated with ethanol through the cAMP‐PKA pathway.

## Graphical abstract

Mallotus furetianus extract (MF) suppressed the development of alcoholic liver fibrosis. MF suppressed the alcohol‐induced activation of hepatic stellate cells. MF suppressed the increase in the activity of NADPH oxidase in HSCs activated with ethanol through the cAMP‐PKA pathway.

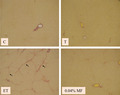

## 1 INTRODUCTION

Alcohol drinking has played an important role in human culture. However, excessive drinking is known to cause alcoholic liver disease (ALD) (Bataller & Brenner, 2005). The first and common change in liver caused by excessive drinking is hepatic steatosis (You & Arteel, 2019). Furthermore, excessive drinking progresses hepatic steatosis to hepatitis, liver fibrosis, cirrhosis, and then hepatocellular carcinoma (Lieber, 1997; Lucey, Mathurin, & Morgan, 2009; Tsukamoto & Lu, 2001). In this process, hepatic steatosis is reversible by timely treatment. Therefore, it is important to find the treatment by which hepatic steatosis returns to normal liver.

*Mallotus furetiamus* is a tropical plant observed in the Hainan Island, China (Lin & Zhou, 1992). Its leaves, commonly called “Shan Ku Cha,” have been drunk as popular aromatic beverage and used as a folk medicine for cholecystitis. The extract also has activities of anti‐oxidation and anti‐atherosclerosis (Liu, Wang, Wu, Qu, & Lin, 2008). Recently, Huang et al. (2017) have shown that the extract of *Mallotus furetiamus* (MF) with hot water decreased the intracellular lipid accumulation in oleic acid‐induced steatosis in hepatocellular carcinoma cells, a cellular hepatic steatosis model. From these results, it is suggested that MF is effective for the treatment of hepatic steatosis in ALD.

The animal models of ALD are important for research of the evaluation for the treatment employed for this disease. For animal models to study ALD rodents are the most suitable model and are the most commonly used (Brandon‐Warnere, Schrum, Scmidt, & McKillop, 2012).

Currently, two animal models for the administration of alcohol, the Lieber–DeCarli liquid diet model (Lieber, De Carli, & Sorrel, 1989) and the Tsukamoto‐French gastric model (Tsukamoto et al., 1995), have been used. However, both models do not bring about cirrhosis in rats. Moreover, Tipoe et al. examined the combined these two diets and showed that an increase in mediators of profibrogenesis was not equally with the histological evidence of fibrosis (Tipoe et al., 2008). On the other hand, Siegers, Pauli, Korb, and Younes (1986) showed that the intrapritonealy injection of low‐dose carbon tetrachloride (CCl_4_) and drinking of a 5% ethanol solution induced experimental fibrosis in rats within 4 weeks. The hepatic histological changes in the ethanol plus CCl_4_‐induced fibrosis rat model and human alcoholic cirrhosis were similar. We also reported the histological change of liver in the ethanol plus CCl_4_‐induced fibrosis model (Kojima‐Yuasa et al., 2003, 2017; Tamura et al., 2013).

In the progression of ALD, reactive oxygen species (ROS) play a key role (Crosas‐Molist & Fabregat, 2015). ROS promotes necrosis and/or apoptosis of hepatocytes and also contributes to liver fibrosis by the increase in the release of pro‐fibrotic cytokines and the expression of collagen gene in hepatic stellate cells (HSCs). When liver damage occurs, HSCs are activated and produce α‐smooth muscle actin and type I collagen (Cui et al., 2011). Then, HSCs change the phenotype to differentiate to the myofibroblasts (Friedman, 2008). Recently, we established an in vitro model of ethanol‐induced injury with HSC and reported that the treatment of *Ecklonia cava* polyphenol prevents HSC activation (Takahashi et al., 2012).

In this study, the protective effect of MF against ALD was examined in in vivo ethanol plus CCl_4_‐induced cirrhosis rat model and in vitro alcohol‐injury model of HSCs.

## 2 MATERIALS AND METHODS

### 2.1 Extraction of *Mallotus furetianus*

Dried *Mallotus furetianus* (Lot No. 20110424044) was purchased from Hainan Ecological Green Tea Limited in China. A voucher specimen (CHN‐Hainan‐201109(001)) has been deposited at The Department of Pharmacognosy, School of Pharmacy, Kitasato University. *Mallotus furetianus* was extracted 3 times with hot water. After filtration, the solvent was evaporated and then lyophilized to dryness.

### 2.2 Animals

The study was accepted by the Osaka City University animal experiment committee (approval number: 1905017) and conducted in accordance with the regulations on animal experiments in Osaka City University. Male Wistar rats from Japan SLC, Inc., Shizuoka, Japan, were housed at 24 ± 1°C with humidity of 40%–60% with 12/12‐hr light and dark cycle. The rats were given water and standard rat chow (LaboMR stock, Japan SLC, Inc.) ad libitum.

### 2.3 Animal experiments

Male Wistar rats (180–210 g body weight) were fed an AIN93G‐based control diet for 5 days. After then, the 30 rats were divided into 5 groups based on body weight by persons not directly related to the experiment.

Group 1: control rats (C);

Group 2: rats given to ethanol plus CCl_4_ (0.1 ml/kg of body weight) (ET);

Group 3 rats given to CCl_4_ (T);

Group 4: rats given to ethanol plus CCl_4_ and 0.012% MF (0.012% MF);

Group 5: rats given to ethanol plus CCl_4_ and 0.04% MF (0.04% MF).

The CCl_4_ solution was given by intraperitoneal injection twice a week (Monday and Thursday), and 5% ethanol in water was given ad libitum for three weeks. In the morning of after three weeks, all the five groups of rats were sacrificed with isoflurane inhalation anesthesia and blood removal with a syringe from the heart.

### 2.4 Histological analysis

Liver tissues of all rats were fixed with 10% buffered formalin fixative. Paraffin‐embedded pathological tissue sections were stained with Weigert's elastic Van Gieson stain. The pathologist was not informed of the detail of the groups.

### 2.5 Liver damage biomarkers

Alanine aminotransferase (ALT) and aspartate aminotransferase (AST) activities in serum of all rats ware assayed with a Transaminase CII‐Test Kit (Fujifilm Wako Pure Chemical Co.).

### 2.6 Preparation and culture of HSCs

Hepatic stellate cells isolated from male Wistar rats (300–350 g body weight) were incubated in Dulbecco's modified Eagle's medium (DMEM) with 10% fetal bovine serum for 3 days (Kojima‐Yuasa et al., 2003). Then, HSCs were incubated in DMEM containing 100 mM of ethanol and with or without 12.5 µg/ml MF.

### 2.7 Lipid peroxidation

The lipid peroxidation was evaluated by the measurement of the thiobarbituric acid‐reactive species (TBARS) (Ohkawa, Onishi, & Yagi, 1979) as following. One hundred microliters of liver homogenate of all rats was incubated with 750 µl of thiobarbituric acid (TBA) (0.8%), 100 µl of sodium dodecyl sulfate (SDS, 8.1%), 750 µl of acetic acid buffer (2.5 M, pH 3.4), and 300 µl of distilled water for 20 min in boiling water. The absorbance was measured spectrophotometrically at 532 nm. The data were showed as nmol equivalent of malondialdehyde (MDA) using tetramethoxypropane as a standard.

### 2.8 Hepatic glutathione (GSH) level

The livers of all rats were homogenated with 9 volumes of 25 mM of Tris–HCl buffer. After homogenization with Polytron PT 1600E and centrifugation, the supernatant treated with 5% perchloric acid (PCA) was analyzed by HPLC. Hepatic GSH level was assayed using 300 µl of the supernatant using the method of Sack, Willi, and Hunziker (2000). The amount of protein was assayed using the Bradford method (Bradford, 1976).

### 2.9 Intracellular ROS Formation

Intracellular ROS formation was analyzed using 2′,7′‐dichlorodihydrofluorescein diacetate (DCFH‐DA) (Tamura et al., 2013). Briefly, 2.4 mM DCFH‐DA (5 μl) was added to the medium before 30 min of the treatment. After washed with phosphate‐buffered saline (PBS) twice, HSCs were observed under a fluorescence imaging system (FSX100 Bio Imaging Navigator, Olympus Corporation). The fluorescence intensity of intracellular ROS was measured by ImageJ.

### 2.10 Immunocytochemistry

HSCs incubated were fixed with 4% paraformaldehyde fixative. Hydrogen peroxide solution and normal goat serum were used for the blocking. Then, the specimens were incubated with an α‐SMA monoclonal antibody or an antitype I collagen polyclonal antibody for primary antibody, and then biotinylated anti‐mouse or anti‐rabbit goat immunoglobulin for second antibody. The specimens were finally incubated with a horseradish peroxidase‐labeled streptavidin‐biotin complex and developed color by 3,3‐diaminobenzidine tetrahydrochloride/nickel chloride. The immunocytochemical intensity of type I collagen or α‐SMA was measured by ImageJ.

### 2.11 Fluorescent immunostaining

HSCs were fixated with 4% paraformaldehyde fixative and then were incubated with anti‐phospho‐Akt (Ser473) polyclonal antibody for primary antibody and goat ant‐rabbit IgG H&L (Alexa Fluor^®^ 488) antibody for secondary antibody. The cells were observed using FSX100 Bio Imaging Navigator. The immunofluorescence intensity of phospho‐Akt was measured by ImageJ.

### 2.12 Statistical analysis

The results are presented as the mean ± *SE*. Significant difference in assay values was performed using analysis of variance followed by Tukey's test. A value of *p* < .05 was considered statistically significant.

## 3 RESULTS

### 3.1 Effect of MF treatment on plasma AST and ALT activities and liver fibrosis

The body weights of ethanol plus CCl4‐treated rats were significantly lowered compared with control group. However, the body weights among ethanol plus CCl_4_‐treated rats, ethanol plus CCl_4,_ and 0.012% MF group and ethanol plus CCl_4_ and 0.04% MF group were not different significantly (Table 1).

**TABLE 1:** The body weight of each group

Groups	Initial body weight (g)	Final body weight (g)
Control	193.24 ± 2.45	303.39 ± 4.60 ^a^
Ethanol plus CCl_4_	191.49 ± 4.24	269.95 ± 5.67 ^b^
CCl_4_ alone	192.61 ± 2.35	275.83 ± 4.33 ^b^
Ethanol plus CCl_4_ and 0.012% MF	191.96 ± 1.67	272.06 ± 2.41 ^b^
Ethanol plus CCl_4_ and 0.04% MF	190.26 ± 1.82	271.32 ± 3.67 ^b^

Note

Data are presented as the mean ± S.E. Values without a common letter are significantly different (*p* < .05).

**FIGURE 1:**
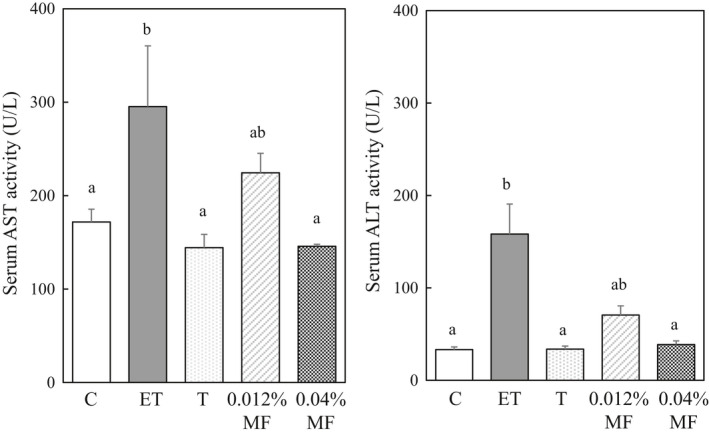
Effect of MF treatment on serum AST and ALT activities and liver fibrosis in ethanol plus CCl_4_‐treated rats. Abbreviations of groups were described in MATERIALS AND METHODS. Data are shown as mean ± *SE* (*n* = 6). Values without a common letter are significantly different (*p* < .05)

After 3 weeks, the plasma AST and ALT activities in rats given with ethanol plus CCl_4_ increased by 1.72‐ and 4.76‐fold, respectively. These enzyme activities in rats given with the ethanol plus CCl_4_ and 0.012% MF showed a decreasing tendency from those in rats given with the ethanol plus CCl_4._ Furthermore, these enzyme activities in the rats given with the ethanol plus CCl_4_ and 0.04% MF were significantly reduced to the levels of the control group rats (Figure 1) suggesting that the protective effect of MF against liver injury with ethanol plus CCl_4_ is dose‐dependent.

**FIGURE 2:**
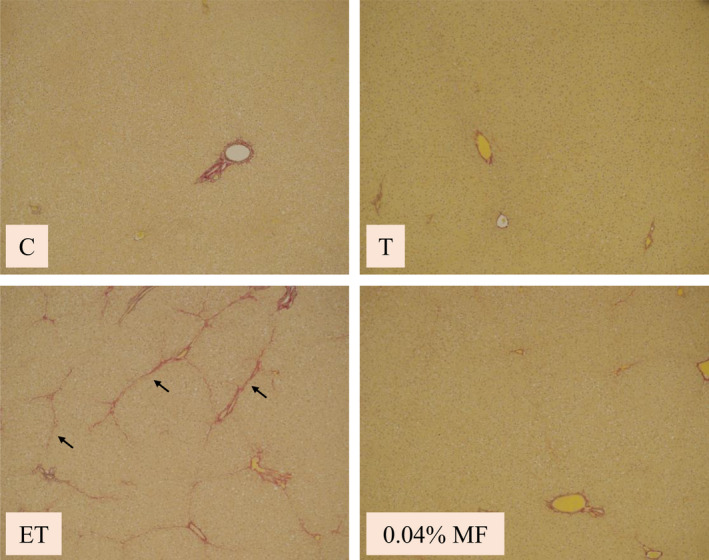
Effect of MF on the changes to liver morphology. Liver sections were processed for Weigert's elastic Van Gieson staining. Abbreviations of groups were described in MATERIALS AND METHODS. Arrows indicate the fibrous septa

Livers of the control rats, MF‐treated rats, and CCl_4_‐treated rats were not observed histological abnormalities. However, lipid accumulation and liver fibrosis were observed in the livers of the ethanol plus CCl_4_‐treated rats. However, ethanol plus CCl_4_‐induced liver steatosis and fibrosis were not observed by the treatment of MF (Figure 2).

### 3.2 Effect of MF treatment on hepatic TBARS and GSH levels in ethanol plus CCl_4_‐treated rats

**FIGURE 3:**
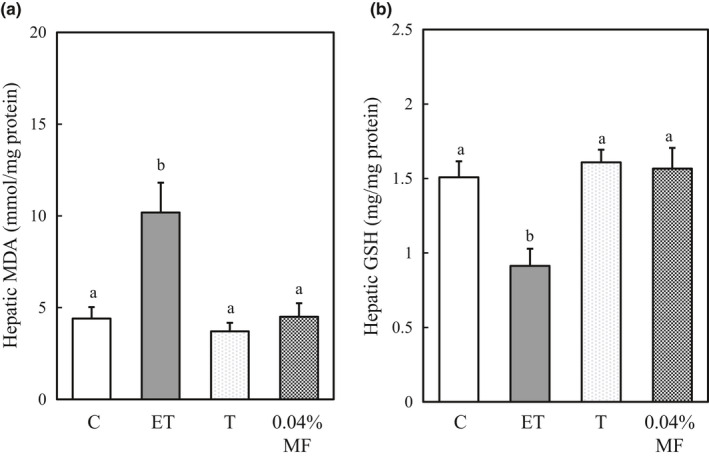
Effect of MF treatment on hepatic TBARS (a) and GSH (b) levels in ethanol plus CCl_4_‐treated rats. Data are presented as the mean ± *SE.* Values without a common letter are significantly different (*p* < .05). Abbreviations of groups were described in MATERIALS AND METHODS

The hepatic TBARS levels of rats treated with ethanol plus CCl_4_ markedly increased as compared to that of the control group rats. However, MF treatment maintained the TBARS level at the control level (Figure 3a). On the other hand, treatment of ethanol plus CCl_4_ significantly lowered the GSH level as compared to control, but MF treatment maintained the GSH level to the control level (Figure 3b).

### 3.3 **Effect of MF on the type I collagen and **α**‐SMA expression in ethanol‐treated HSCs**

**FIGURE 4:**
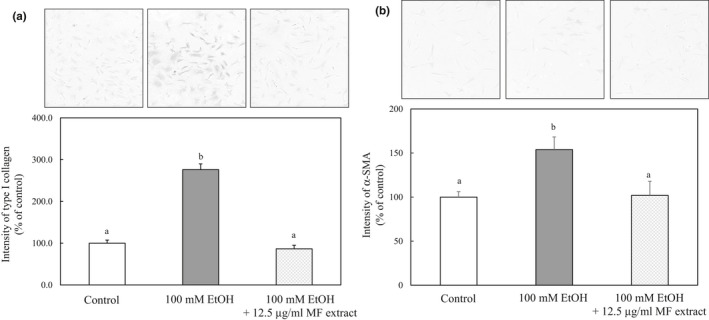
Effect of MF on the type I collagen (a) and α‐SMA (b) expression in ethanol‐treated HSCs. HSCs were incubated for 24 hr with 100 mM of ethanol and with or without 12.5 µg/ml of MF. The immunocytochemical intensity of type I collagen or α‐SMA was measured by ImageJ. Data are presented as the mean ± *SE*. Values without a common letter are significantly different (*p* < .01)

The effect of MF on the type I collagen and α‐SMA expression was examined by immunohistochemistry in ethanol‐treated HSCs. As shown in Figure 4, the ethanol‐induced increases in type I collagen and α‐SMA expression were suppressed to near‐control levels by the treatment of MF.

### 3.4 Effect of MF on intracellular ROS levels in ethanol‐treated HSCs

**FIGURE 5:**
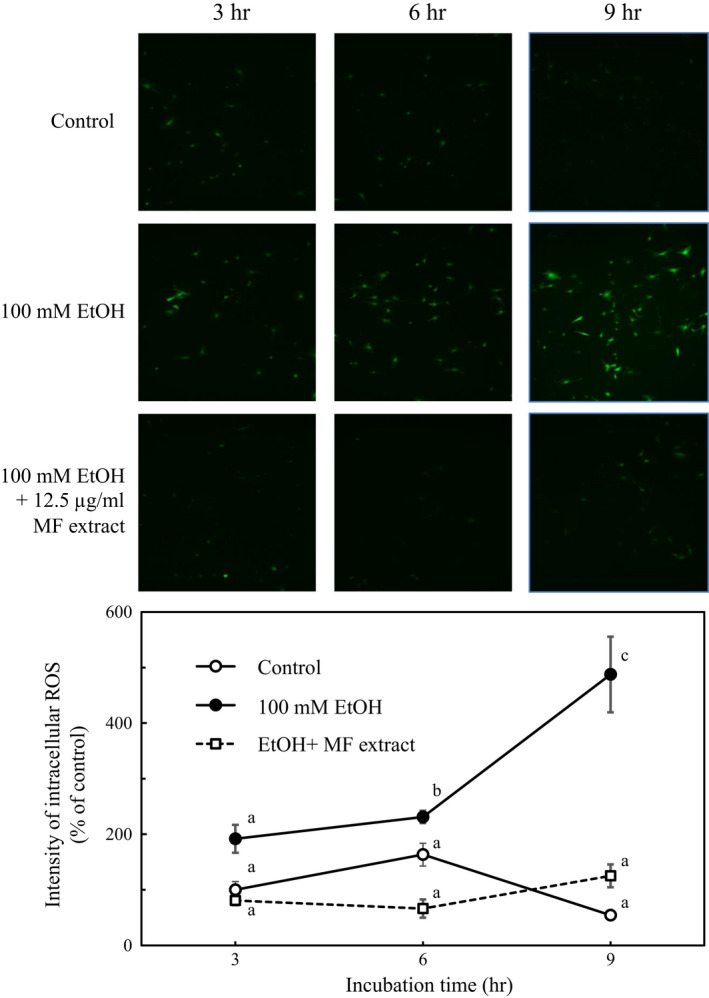
Effect of MF on intracellular ROS levels in ethanol‐treated HSCs. HSCs were incubated for 3, 6, or 9 hr with 100 mM of ethanol, with or without 12.5 µg/ml of MF. The fluorescence intensity of intracellular ROS was measured by ImageJ. Data are presented as the mean ± *SE*. Values without a common letter are significantly different (*p* < .01)

The intracellular ROS levels were measured using DCF‐DA, which is converted to the highly fluorescent 2’,7‐dichlorodihydrofluoresscein in the presence of intracellular ROS. HSCs incubated for 3, 6, and 9 hr with 100 mM of ethanol enhanced the intracellular ROS significantly. However, MF treatment maintained intracellular ROS levels to the levels of the control cells (Figure 5).

### 3.5 Effect of diphenyleneiodonium (DPI) on intracellular ROS levels and on the expressions of type I collagen and α‐SMA in ethanol‐treated HSCs

**FIGURE 6:**
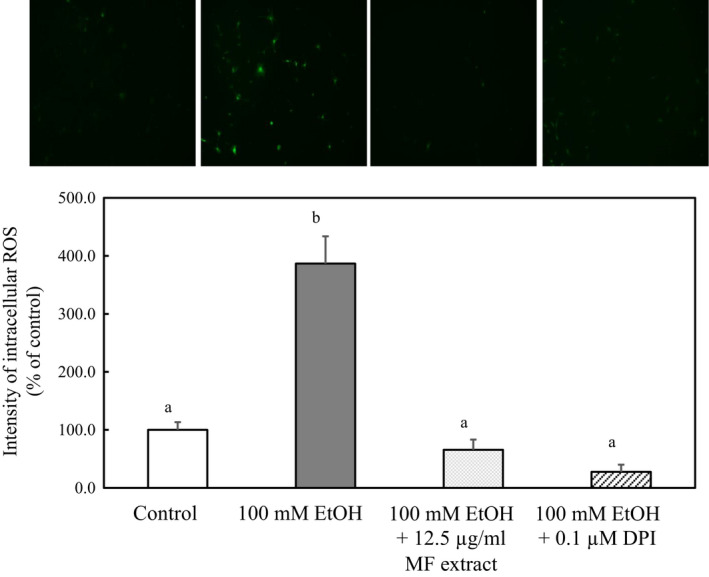
Effect of DPI on intracellular ROS levels in ethanol‐treated HSCs. HSCs were incubated for 9 hr with 100 mM of ethanol, with or without 12.5 µg/ml of MF or 0.1 μM of DPI. The fluorescence intensity of intracellular ROS was measured by ImageJ. Data are presented as the mean ± *SE*. Values without a common letter are significantly different (*p* < .01)

We examined whether ethanol‐induced ROS were mainly produced by nicotinamide adenine dinucleotide phosphate (NADPH) oxidase in HSCs. Cells were treated with NADPH oxidase inhibitor DPI (0.1 µM) and ethanol (100 mM) for 9 hr. The treatment of DPI suppressed the intracellular ROS levels enhanced with ethanol to the levels of the control cells (Figure 6). These results indicated that NADPH oxidase inhibitor DPI inhibited the generation of ROS induced by ethanol, suggesting that NADPH oxidase could be a source of ROS in HSCs.

**FIGURE 7:**
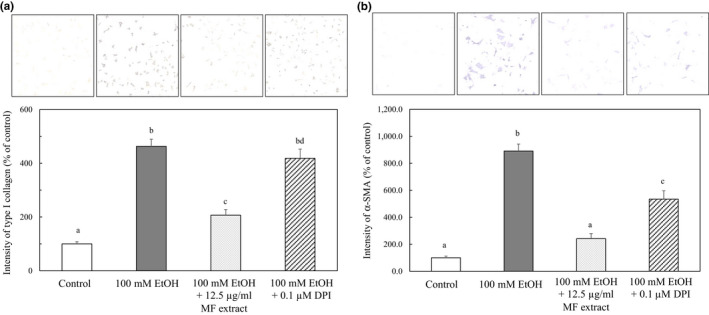
Effect of DPI on the type I collagen (a) and α‐SMA (b) expression in ethanol‐treated HSCs. HSCs were incubated for 24 hr with 100 mM of ethanol and with or without 12.5 µg/ml of MF or 0.1 μM of DPI. The immunocytochemical intensity of type I collagen or α‐SMA was measured by ImageJ. Data are presented as the mean ± *SE*. Values without a common letter are significantly different (*p* < .01)

Furthermore, we treated HSCs with DPI and ethanol for 24 hr, and we examined the effect of DPI on the expression of type I collagen and α‐SMA by immunohistochemistry. As shown in Figure 7, the ethanol‐induced increases in type I and α‐SMA expression were suppressed by the treatment of DPI to near‐control levels. These results suggested that NADPH oxidase regulates the activation of HSCs through the ROS formation.

### 3.6 Effect of MF on the PI3K‐Akt pathway in ethanol‐treated HSCs

**FIGURE 8:**
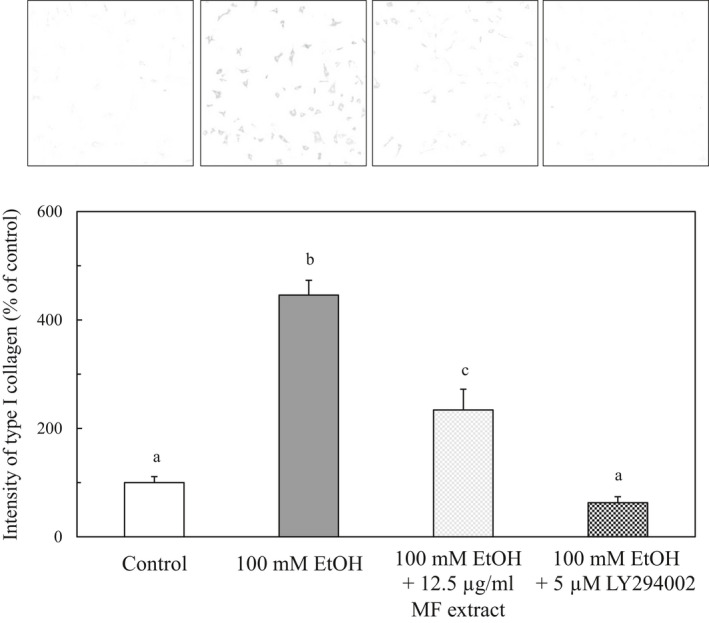
Effect of MF or LY294002, an inhibitor of PI3K, on the type I collagen expression in ethanol‐treated HSCs. HSCs were incubated for 24 hr with 100 mM of ethanol and with or without 12.5 µg/ml of MF or 5 μM LY294002s. The immunocytochemical intensity of type I collagen was measured by ImageJ. Data are presented as the mean ± *SE*. Values without a common letter are significantly different (*p* < .01)

Son, Hines, Lindquist, Schrum, and Rippe (2009) reported that the inhibition of PI3K signaling during the activation of HSCs inhibited the synthesis of type I collagen and reduced the expression of profibrogenic factors. Lu et al. (2010) have also shown that the PI3K‐Akt pathway regulates proliferation and collagen production in bleomycin‐induced fibroblast. Therefore, the effect of LY294002, an inhibitor of PI3K, on expression of type I collagen was examined in ethanol‐treated HSCs. The ethanol‐induced increase in type I collagen expression in HSCs was suppressed by the treatment of LY194002 (Figure 8), suggesting that ethanol induces expression of type I collagen through upregulation of the PI3K‐Akt pathway.

**FIGURE 9:**
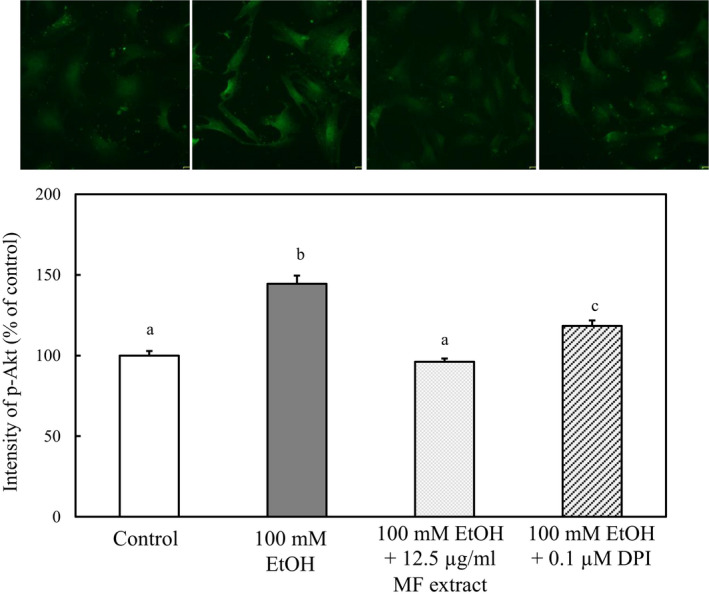
Effect of MF on the phosphorylation of Akt in ethanol‐treated HSCs. HSCs were incubated for 24 hr with 100 mM of ethanol and with or without 12.5 µg/ml of MF or 0.1 µM of DPI. The immunofluorescence intensity of phospho‐Akt was measured by ImageJ. Data are presented as the mean ± *SE*. Values without a common letter are significantly different (*p* < .01)

Furthermore, the effect of MF and DPI on phosphorylation of AKT was investigated. As shown in Figure 9, the increase in the levels of phosphorylated Akt in ethanol‐treated HSCs was suppressed with the treatment of MF or DPI.

### 3.7 Effect of MF and H‐89, an inhibitor of PKA, on expression of type I collagen in ethanol‐treated HSCs

**FIGURE 10:**
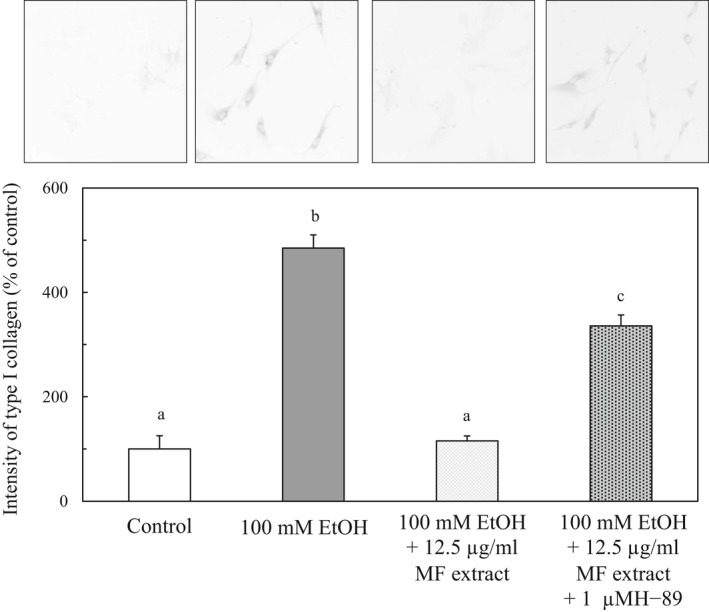
Effect of MF and H‐89, an inhibitor of PKA, on the type I collagen expression in ethanol‐treated HSCs. HSCs were incubated for 24 hr with 100 of mM ethanol and with or without 12.5 µg/ml of MF or 1 μM of H‐89. The immunocytochemical intensity of type I collagen was measured by ImageJ. Data are presented as the mean ± *SE*. Values without a common letter are significantly different (*p* < .01)

Mallat et al. (1998) have reported that the increase in cAMP activates cAMP‐response element binding through the activation of cAMP‐dependent protein kinase (PKA) and then inhibits collagen synthesis and α‐SMA in human HSCs. The effect of H‐89, an inhibitor of PKA, on the collagen synthesis in HSCs treated with ethanol and MF was examined. As shown in Figure 10, the increase in the collagen synthesis was suppressed with the treatment of MF. However, the suppression of the synthesis of type I collagen in MF‐treated HSCs was inhibited by the addition of 1 μM of H‐89, suggesting that the MF‐induced inhibition against the activation of HSCs with ethanol is dependent on the cAMP‐PKA pathway.

## 4 DISCUSSION

The present study demonstrated that MF protected against ethanol plus CCl_4_‐induced liver injury in rats dose‐dependently. The increases in type I collagen and α‐SMA expression in the livers of ethanol plus CCl_4_‐induced rats were suppressed with the treatment of MF by keeping intracellular ROS and glutathione levels. Furthermore, we evaluated the effect of MF on the HSCs activation, which is responsible for the increased production and deposition of the extracellular matrix in liver injury to determine the detailed mechanism of the protective effect of MF. Activation of HSCs includes the increased the expression of type I collagen, the expression of cytoskeleton markers, such as α‐SMA, and increased proliferation (Friedman, 2000).

ROS are an important trigger for HSC activation (Svegliati‐Baroni et al., 2001). In the present study, we also observed the enhancement of the intracellular ROS levels and the increase in the expressions of type I collagen and α‐SMA in HSCs activated with ethanol. Furthermore, the enhancement of ROS levels was suppressed with the treatments of MF or DPI, an inhibitor of NADPH oxidase. These results suggest that NADPH oxidase could be a source of ROS in HSCs activated with ethanol. MF or DPI treatments also suppressed the ethanol‐induced increase in the expressions of type I collagen and α‐SMA.

The PI3K‐Akt signaling pathway may regulate the activation of HSCs (Son et al., 2009). We observed that the increase in the expression of type I collagen in HSCs activated with ethanol was suppressed by an inhibitor of PI3K, suggesting that ethanol induced type I collagen expression via the PI3K‐Akt signaling pathway. On the other hand, the treatments with MF or DPI suppressed the phosphorylation of Akt in HSCs activated with ethanol, suggesting that ROS increased the phosphorylation of Akt in the HSCs.

It is reported that **t**he pathways of cAMP and PKA regulate the synthesis of collagen synthesis in human HSCs (Lopet‐Sanchez et al., 2014). We observed that the suppression of the synthesis of type I collagen in MF‐treated HSCs was inhibited by the addition of a PKA inhibitor. From these results, the cAMP‐PKA pathway may be involved in the MF‐induced inhibition against the activation of HSCs with ethanol. In regard to the relationship between NADPH oxidase and the cAMP‐PKA pathway, Qiao et al. (2017) have observed that intermedin alleviates unilateral ureteral obstruction‐induced renal fibrosis by inhibition of ROS, and in this model, the activity of NADPH oxidase is regulated with the pathway of cAMP and PKA. From these results, MF may cause an activation of the cAMP and PKA pathways, and then, the pathway suppressed the increase in the activity of NADPH oxidase in HSCs activated with ethanol.

Huang et al. (2017) isolated 8 compounds and found that 3 compounds, (Z)‐3‐hexenyl‐β‐D‐glucopyranoside, (+)‐lyoniresinol‐3 α‐*O*‐ α‐Lrhamnopyranoside, and mallophenol A, had antisteatosis activity in the steatosis cell model. However, the protective effect of MF on alcohol‐induced liver injury in an in vivo rat model and an in vitro model of HSCs is unclear because the evaluations in these experimental systems require large samples. This should be clarified in future studies.

In conclusion, these results strongly suggest that MF is beneficial in the prevention of ethanol‐induced liver injury. However, further studies should examine the clinical effect of MF to more fully understand its potential.

## 5 ETHICAL REVIEW

The study was approved by the Osaka City University animal experiment committee (approval number: 1905017) and conducted in accordance with the regulations on animal experiments in Osaka City University.

## CONFLICT OF INTEREST

The authors declare that they have no conflict of interest.

## References

[fsn31709-bib-0001] Bataller, R. , & Brenner, D. A. (2005). Liver fibrosis. Journal of Clinical Investigation, 115, 209–218. 10.1172/JCI24282 15690074 PMC546435

[fsn31709-bib-0002] Bradford, M. M. (1976). A rapid and sensitive method for the quantitation of microgram quantities of protein utilizing the principle of protein‐dye binding. Analytical Biochemistry, 72, 248–254. 10.1016/0003-2697(76)90527-3 942051

[fsn31709-bib-0003] Brandon‐Warnere, E. , Schrum, L. W. , Scmidt, C. M. , & McKillop, I. H. (2012). Rodent models of alcoholic liver disease of mice and men. Alcohol, 46, 715–725. 10.1016/j.alcohol.2012.08.004 22960051 PMC3496818

[fsn31709-bib-0004] Crosas‐Molist, E. , & Fabregat, I. (2015). Role of NADPH oxidases in the redox biology of liver fibrosis. Redox Biology, 6, 106–111. 10.1016/j.redox.2015.07.005 26204504 PMC4804101

[fsn31709-bib-0005] Cui, W. , Matsuno, K. , Iwata, K. , Ibi, M. , Matsumoto, M. , Zhang, J. , … Torok, N. J. (2011). Yabe‐Nishimura C. NOX1/nicotinamide adenine dinucleotide phosphate, reduced form (NADH) oxidase promotes proliferation of stellate cells and aggravates liver fibrosis induced by bile duct ligation. Hepatology, 54, 949–958. 21618578 10.1002/hep.24465

[fsn31709-bib-0006] Friedman, S. L. (2000). Molecular regulation of hepatic fibrosis, an integrated cellular response to tissue injury. Journal of Biological Chemistry, 275, 2247–2250. 10.1074/jbc.275.4.2247 10644669

[fsn31709-bib-0007] Friedman, S. L. (2008). Hepatic stellate cells; protean, multifunctional, and enigmatic cells of the liver. Physiologycal Reviews, 88, 125–172. 10.1152/physrev.00013.2007 PMC2888531 18195085

[fsn31709-bib-0008] Huang, X. , Xu, M. , Shirahata, T. , Li, W. , Koike, K. , Kojima‐Yuasa, A. , … Kobayashi, Y. (2017). Anti‐steatosis compounds from leaves of *Mallotus furetianus* . Natural Product Research, 32:1459–1462. 10.1080/14786419.2017.1350664 28693358

[fsn31709-bib-0009] Kojima‐Yuasa, A. , Hirauchi, E. , Sawada, T. , Kasahara, A. , Yoshikawa, E. , Tamura, A. , & Matsui‐Yuasa, I. (2017). Extract of traditional pickling melon prevents ethanol‐induced liver injury in rats. International Journal of Food and Nutritional Science, 6, 56–66.

[fsn31709-bib-0010] Kojima‐Yuasa, A. , Ohkita, T. , Yukami, K. , Ichikawa, H. , Takami, N. , Nakatani, T. , … Matsui‐Yuasa, I. (2003). Involvement of intracellular glutathione in zinc deficiency‐induced activation of hepatic stellate cells. Chemico‐Biological Interactions, 146, 89–99. 10.1016/S0009-2797(03)00087-5 12902156

[fsn31709-bib-0011] Lieber, C. S. (1997). Ethanol metabolism, cirrhosis and alcoholism. Clinica Chimica Acta, 257, 59–84. 10.1016/S0009-8981(96)06434-0 9028626

[fsn31709-bib-0012] Lieber, C. S. , De Carli, L. M. , & Sorrel, M. F. (1989). Experimental methods of ethanol administration. Hepatology, 10, 501–510. 10.1002/hep.1840100417 2673971

[fsn31709-bib-0013] Lin, H. , & Zhou, S. (1992). The biological character and ecological distribution of *Mallotus furetianus* in Hainan. Natural Science Journal of Hainan University, 10, 32–34.

[fsn31709-bib-0014] Liu, Y. L. , Wang, L. Q. , Wu, H. T. , Qu, X. , & Lin, L. B. (2008). Effect of *Mallotus furetianun* on preventive development of atherosclerosis in atherosclerotic rats. Journal of Hainan Medical College, 14, 608–611.

[fsn31709-bib-0015] Lopet‐Sanchez, I. , Dunkel, Y. , Roh, Y. S. , Mittal, Y. , De Minicis, S. , Muranyi, A. , … Ghosh, P. (2014). GIV/Girdin is a central hub for profibrogenic signaling networks during liver fibrosis. Natural Communications, 2014(5), 445 10.1038/ncomms5451 PMC4107319 25043713

[fsn31709-bib-0016] Lu, Y. , Azad, N. , Wang, L. , Iyer, A. K. V. , Castranova, V. , Jiang, B. H. , & Rojansakul, Y. (2010). Phosphatidylinositol‐3‐kinase/Akt regulates bleomucin‐induced fibroblast proliferation and collagen production. American Journal of Respiratory Cell and Molecular Biology, 42, 432–441. 19520917 10.1165/rcmb.2009-0002OC PMC2848736

[fsn31709-bib-0017] Lucey, M. R. , Mathurin, P. , & Morgan, T. R. (2009). Alcoholic hepatitis. New England Journal of Medicine, 360, 2758–2769. 10.1056/NEJMra0805786 19553649

[fsn31709-bib-0018] Mallat, A. , Gallois, C. , Tao, J. , Habib, A. , Maclouf, J. , Mavier, P. , … Lotersztain, S. (1998). Platelet‐derived growth factor‐BB and thrombin generate positive and negative signals for human hepatic stellate cell proliferation: Role of a prostaglandin/cyclic AMP pathway and cross‐talk with endothelin receptors. Journal of Biological Chemistry, 273, 27300–27305. 9765255 10.1074/jbc.273.42.27300

[fsn31709-bib-0019] Ohkawa, H. , Onishi, N. , & Yagi, K. (1979). Assay for lipidperoxide in animal tissues by thiobarbituric acid reaction. Analytical Biochemistry, 95, 351–358. 36810 10.1016/0003-2697(79)90738-3

[fsn31709-bib-0020] Qiao, X. , Wang, L. , Wang, Y. , Su, X. , Qi, Y. , Fan, Y. , & Peng, Z. (2017). Intermedin inhibits unilateral ureteral obstruction‐induced oxidative stress via NADPH oxidase Nox4 and cAMP‐dependent mechanism. Renal Failure, 39, 652–659. 28805491 10.1080/0886022X.2017.1361839 PMC6447914

[fsn31709-bib-0021] Sack, R. A. , Willi, A. , & Hunziker, P. E. (2000). Determination of total glutathione in cell lysates by high performance liquid chromatography with O‐phthalaldehyde precolumn derivatization in the presence of tris(1‐carboxyethyl)‐phosphate. Journal of Liquid Chromatography & Related Technologies, 23, 2947–2962.

[fsn31709-bib-0022] Siegers, C. P. , Pauli, V. , Korb, G. , & Younes, M. (1986). Hepatoprotection by malotilate against carbon tetrachloride‐alcohol‐induced liver fibrosis. Agents and Actions, 18, 600–602. 10.1007/BF01964970 3766314

[fsn31709-bib-0023] Son, G. , Hines, I. N. , Lindquist, J. , Schrum, L. W. , & Rippe, R. A. (2009). Inhibition of phosphatidylinositol 3‐kinase signaling in hepatic stellate cells blocks the progression of hepatic fibrosis. Hepatology, 50, 1512–1523. 10.1002/hep.23186 19790269 PMC2913293

[fsn31709-bib-0024] Svegliati‐Baroni, G. , Saccomanno, S. , van Goor, H. , Jansen, P. , Benedetti, A. , & Moshage, H. (2001). Involvement of reactive oxygen species and nitric oxide radicals in activation and proliferation of rat hepatic stellate cells. Liver, 21, 1–12. 10.1034/j.1600-0676.2001.210101.x 11169066

[fsn31709-bib-0025] Takahashi, M. , Satake, N. , Yamoshita, H. , Tamura, A. , Sasaki, M. , Matsui‐Yuasa, I. , … Kojima‐Yuasa, A. (2012). Ecklonia cava polyphenol protects the liver against ethanol‐induced injury in rats. Biochimica et Biophysica Acta, 1820, 978–988. 10.1016/j.bbagen.2012.02.008 22387226

[fsn31709-bib-0026] Tamura, A. , Sasaki, M. , Yamashita, H. , Matsui‐Yuasa, I. , Saku, T. , Hikima, T. , … Kojima‐Yuasa, A. (2013). Yerba‐mate (*Ilex paraguarienesis*) extract prevents ethanol‐induced liver in rats. Journal of Functional Foods, 5, 1714–1723.

[fsn31709-bib-0027] Tipoe, G. L. , Liong, E. C. , Casey, C. A. , Donohue, T. M. Jr , Eagon, P. K. , So, H. , … Nanji, A. A. (2008). A voluntary oral ethanol‐feeding rat model associated with necroinflammatory liver injury. Alcoholism: Clinical and Experimental Research, 32, 669–682. 10.1111/j.1530-0277.2008.00623.x 18341647

[fsn31709-bib-0028] Tsukamoto, H. , Horne, W. , Kamimura, S. , Niemela, O. , Parkkila, S. , Ylaherttuala, S. , & Brittenham, G. M. (1995). Experimental liver‐cirrhosis induced by alcohol and iron. Journal of Clinical Investigation, 96, 620–630. 10.1172/JCI118077 7615836 PMC185237

[fsn31709-bib-0029] Tsukamoto, H. , & Lu, S. C. (2001). Current concept in the pathogenesis of alcoholic liver injury. FASEB Journal, 15, 1335–1349. 11387231 10.1096/fj.00-0650rev

[fsn31709-bib-0030] You, M. , & Arteel, G. E. (2019). Effect of ethanol on lipid metabolism. Journal of Hepatology, 70, 237–248. 10.1016/j.jhep.2018.10.037 30658725 PMC6436537

